# Supplementary material for: Burnout and sleep problems among nurses working in a tertiary hospital in Kathmandu, Nepal
Source: PLOS Glob Public Health. 2025 Jul 11;5(7):e0003879. doi: 10.1371/journal.pgph.0003879 (PMC12250318; doi:10.1371/journal.pgph.0003879)
Supplement: Attachment — Submitted filename: Burnout Reviewers Comment_Clinton Niba.docx [file pgph.0003879.s002.docx]

**Reviewer:** Niba, Clinton Ambe

**Paper Title:** Burnout and sleep problems among nurses working in a tertiary hospital in Kathmandu, Nepal

**Reviewer Comments:**

**INTRODUCTION**

- Nicely written introduction. However, why should we care if nurses' burnout is not directly affecting the care recipients (patients)? Anyone can experience burnout. It would be helpful to emphasize how stress and burnout among nurses have affected the care provided to patients. For instance, in line 46 & 47 you mentioned burnout resulting to ".....professional failures". What are these failures? Are there reported statistics on how nurses may have failed to act appropriately due to stress or burnout, particularly in Nepal or countries with similar demographics? I recommend this addition to the already nicely written introduction.

- There seem to be a grammatical problem in line 68-71

**RESULTS**

- The results have been nicely presented. I suggest that you briefly present the prevalence of burnout in one sentence before introducing the socio-demographics variables, as this is a crucial finding that readers will be eager to see. Currently, it appears far down in the presentation. You may also include the sample size and response rate in one sentence before presenting the prevalence of burnout.

- Did you by any means thought of considering the association between gender/sex or marital status and experiencing burnout? some studies elsewhere have reported fascinating results on these variables.

**DISCUSSION**

I have read some recent and very similar studies that report burnout among nurses in Nepal. Look and see how helpful this can be in this paper. See

(1) Shrestha et al., (2021), available at: <https://nepjol.info/index.php/IJOSH/article/view/37259>

(2) Shah et al., (2024), available at: <https://pmc.ncbi.nlm.nih.gov/articles/PMC11261546/>

This can prompt you to look again into the literature to avoid duplication of results.

**RECOMMENDATIONS**

To the best of my knowledge, I think it would have been necessary to provide some recommendations to improve nurses’ efficiency and reduce stress and burnout in the workplace. It might not necessarily be a government policy recommendation, but an institutional recommendation that can be incorporated within the Bylaws of the institution or hospital or healthcare organizations.
